# Pancreatic Endocrine Neoplasm Concomitant with a Complicated Endocrine History: A Case Report and Literature Review

**DOI:** 10.1089/pancan.2017.0001

**Published:** 2017-03-01

**Authors:** Amelia Rogers, Christine Lotto, Charles J. Yeo

**Affiliations:** Department of Surgery, Sidney Kimmel Medical College, Thomas Jefferson University, Philadelphia, Pennsylvania.

**Keywords:** atrophic gastritis, gastric carcinoid syndrome, hypergastrinemia, pancreatic neuroendocrine tumor, pernicious anemia

## Abstract

**Background:** Pancreatic neuroendocrine tumors (PNETs) are rare, and metastases when present are most commonly found in the liver or the peripancreatic lymph nodes. In this study, we present a patient who developed a metastatic PNET in the liver in the setting of multiple concomitant autoimmune disorders, including pernicious anemia and atrophic gastritis with hypergastrinemia.

**Case presentation:** The patient is a 70-year-old woman with a history of Hashimoto's thyroiditis, thymoma, gastric carcinoid tumors, and autoimmune atrophic gastritis with pernicious anemia. She was found to have a 2 cm mass in the pancreaticoduodenal groove originating from the pancreas. A preoperative endoscopic ultrasound with fine-needle aspiration showed a well-differentiated PNET. During surgery, she was found to have multiple subcentimeter liver lesions, which on frozen section were shown to be a metastatic neuroendocrine tumor. After surgical resection, final pathology revealed a PNET with metastases to the liver. The metastatic lesions stained positive for gastrin.

**Conclusion:** We were only able to find one other example in the literature of a PNET occurring in association with pernicious anemia. Our patient developed a metastatic PNET in the setting of multiple autoimmune disorders, including pernicious anemia.

## Introduction and Background

Pancreatic neuroendocrine tumors (PNETs) are rare, accounting for <1% to 2% of all pancreatic masses.^1^ In this study, we present a patient who developed a metastatic PNET. She had a history of several other concomitant and rare autoimmune disorders, including autoimmune atrophic gastritis and pernicious anemia. It is known that gastric carcinoid tumors are associated with pernicious anemia, likely stimulated by the associated hypergastrinemia.^2^ Our patient additionally had a history of gastric carcinoid tumors.

## Case Presentation

A young-appearing 70-year-old woman presented with a mass in the head of her pancreas in the setting of multiple known autoimmune diseases. She had a long-standing history of Hashimoto's thyroiditis and a prior thymoma resection. She also had a history of hypergastrinemia and atrophic gastritis, both of which were manifestations of pernicious anemia, as evidenced by anemia associated with a low serum B12 level of 117 pg/mL (normal: 200–350 pg/mL), an equivocal intrinsic factor blocking Ab assay, and an elevated gastric parietal cell antibody titer of 39.5 (positive >25). Because of this, and her lack of any history of peptic ulcer disease, the patient was not thought to have had hypergastrinemia because of the Zollinger–Ellison syndrome. Her pernicious anemia required monthly vitamin B12 injections. She also had several small, gastric fundal polyps followed yearly with esophagogastroduodenoscopy, and she had a history of endoscopic resection of these gastric carcinoid tumors arising in the polyps.

**FIG. 1.**
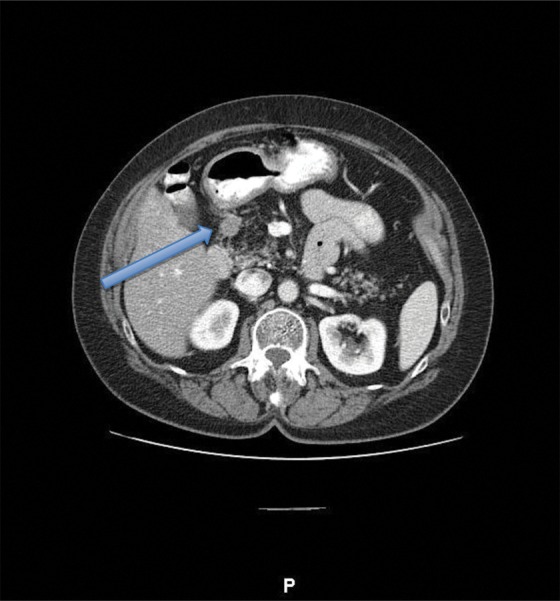
Computed tomography scan showing a 2 cm mass (arrow) in the pancreaticoduodenal groove near D1–D2.

Owing to some vague abdominal pain, she underwent a computed tomography scan that showed a 2 cm mass extending extrinsically from the pancreaticoduodenal groove (Fig. 1). An endoscopic ultrasound confirmed a 17 mm well-circumscribed, hypoechoic mass within the head of the pancreas. Fine-needle aspiration demonstrated a well-differentiated PNET. Upon presentation, she was asymptomatic, without signs of obstructive jaundice or weight loss. Preoperative laboratory results included a chromogranin A level of 20 ng/L (ref <39 ng/L) and a markedly elevated gastrin level of 3484 pg/mL (ref. <100 pg/mL).

Exploration of the abdomen revealed a marble-sized mass just inferior to D1 in the superior head of the pancreas. The remainder of the pancreas was firm and the duodenum was not involved. Assessment of the liver demonstrated myriad pale blue-colored, subcentimeter, cystic-appearing lesions. A frozen section from these lesions in the liver showed metastatic neuroendocrine tumor. The lesion in the superior aspect of the pancreatic head was then dissected and found to be fairly deep in the gland. With distant disease and being wary of the proximity of the mass to the main pancreatic duct, a partial enucleation of the primary pancreatic mass was performed. She did well postoperatively and was discharged home on postoperative day 4. Her further recovery was uneventful.

**FIG. 2.**
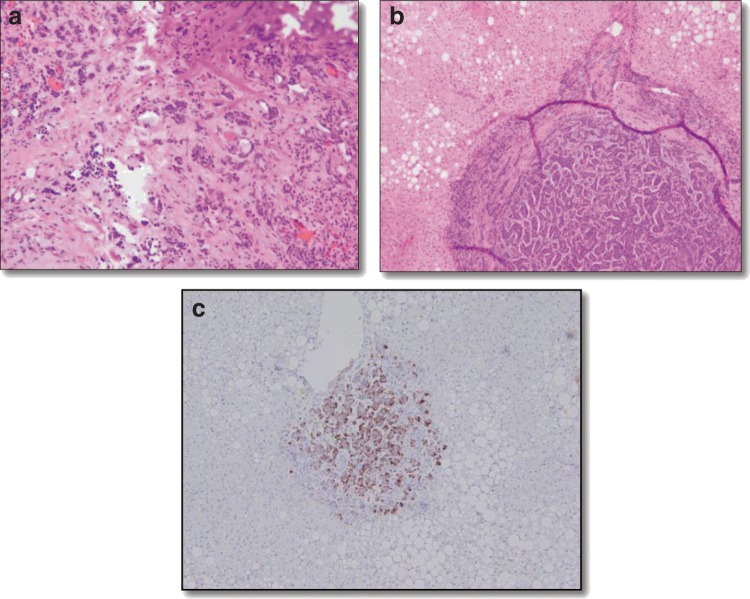
**(a)** H + E stain of the well-differentiated pancreatic neuroendocrine tumor at 20× power; **(b)** H + E stain of liver metastasis at 10× power; and **(c)** Gastrin stain (+) liver metastasis at 20× power. H+E, hematoxylin and eosin stain.

Final pathology confirmed metastatic neuroendocrine tumor in the liver and a primary PNET. The pancreatic tumor stained positive for synaptophysin and CD56, with a Ki67 proliferation index of 10%. Owing to the degree of necrosis of the primary pancreatic tumor, immunohistochemistry staining for gastrin was nonspecific. The liver biopsy showed features similar to the PNET and stained positive by immunohistochemistry for gastrin (Fig. 2).

## Discussion

Carcinoid tumors are relatively rare, with reports describing an annual incidence of 2.0–4.7 out of 100,000 patients.^3,4^ Gastric carcinoid tumors are well-differentiated neuroendocrine tumors that arise from the enterochromaffin-like (ECL) cells of the digestive tract. Gastrin, released by antral G cells of the stomach, stimulates the ECL cells to secrete histamine, which acts on parietal cells to secrete hydrogen ions (acid) to lower the pH of the gastric secretions.^5^ Gastrin also directly stimulates acid secretion by parietal cells. Repeated overstimulation of gastric ECL cells by a hypergastrinemic state is the presumed pathogenesis of gastric carcinoid tumor formation.^2^ Pernicious anemia and the resulting achlorhydria and hypergastrinemia are one such cause for the development of G cell hyperplasia and elevated gastrin levels. At the time of presentation, our patient had a serum gastrin level of 3484 pg/mL (ref. <100 pg/mL), in retrospect perhaps because of both her achlorhydria and her metastatic PNET, and gastrin positive in the liver. Such patients may also have other associated endocrinopathies such as hypothyroidism, diabetes, and hypoadrenocoricoidism. Our patient had a history of thymoma and hypothyroidism from Hashimoto's thyroiditis.

There are many conditions that may cause a patient to have an increase in fasting gastrin level and it can be difficult to distinguish benign causes from the gastrinomas. Two-thirds of patients with gastrinomas had <10-fold increase in gastrin, which overlaps with more common conditions, and <10% have a very high (>100-fold increase) in gastrin.^6^ It must be differentiated whether the hypergastrinemia is associated with a hypo- or achlorhydic state, such as pernicious anemia, atrophic gastritis, gastric cancer, postvagotomy, or antisecretory drug induced, as compared with a hyperchlorhydria, as in *Helicobacter pylori* infection, peptic ulcer disease with obstruction, antral G-cell hyperplasia, renal failure, short bowel syndrome, or Zollinger–Ellison syndrome.^6^ In addition, a gastrin provocation test with secretin has a high sensitivity and specificity for diagnosing patients with gastrinomas.^6^ Our patient had a high gastrin level and did not undergo a gastrin provocation test. She unfortunately likely has both a metastatic gastrinoma and achlorhydic hypergastrinemia because of pernicious anemia.

PNETs arise from islet cells. Up to 50–70% of these tumors are nonfunctioning and do not secrete the hormones of their cells or origin. They have a low annual incidence, <1 out of 100,000, and comprise only about 1% to 2% of all pancreatic tumors.^7^ Although they tend to have a more favorable prognosis than exocrine pancreas cancer, hepatic metastases are observed in more than 50% of patients with PNETs. The 5-year overall survival for patients with PNETs is reported to be 92–100% for stage I and 57% for stage IV disease, depending on the staging system utilized.^8^ Without a strong index of suspicion, it would be easy for a physician to contribute the sole cause of a patient's hypergastrinemia to his or her underlying autoimmune and endocrinopathies. In doing so, they could miss a potentially more aggressive pancreatic exocrine cancer.

Upon our review of the literature, we found only a single case report of a woman with gastric carcinoid tumors in the setting of atrophic gastritis, who subsequently developed a pancreatic endocrine neoplasm.^9^ In this study, we present a patient with a similar history who was found to have a primary PNET with hepatic metastatic disease, in the setting of pernicious anemia, autoimmune atrophic gastritis, and gastric carcinoid tumors. She has been referred to medical oncology and is receiving treatment with small molecule therapy and depo-octreotide, now 3 months postresection.

## Abbreviations Used

PNET: pancreatic neuroendocrine tumor
ECL: enterochromaffin-like
